# Effects of exposure to bodies of different sizes on perception of and satisfaction with own body size: two randomized studies

**DOI:** 10.1098/rsos.171387

**Published:** 2018-05-09

**Authors:** Helen Bould, Rebecca Carnegie, Heather Allward, Emily Bacon, Emily Lambe, Megan Sapseid, Katherine S. Button, Glyn Lewis, Andy Skinner, Matthew R. Broome, Rebecca Park, Catherine J. Harmer, Ian S. Penton-Voak, Marcus R. Munafò

**Affiliations:** 1 Department of Psychiatry, University of Oxford, Oxford, UK; 2 Centre for Academic Mental Health, University of Bristol, Bristol, UK; 3 School of Experimental Psychology, University of Bristol, Bristol, UK; 4 Department of Psychology, University of Bath, Bath, UK; 5 Division of Psychiatry, University College London, London, UK; 6 Institute for Mental Health, University of Birmingham, Birmingham, UK; 7 MRC Integrative Epidemiology Unit at the University of Bristol, Bristol, UK

**Keywords:** body, weight, body dissatisfaction, eating disorders, perception, body size

## Abstract

Body dissatisfaction is prevalent among women and associated with subsequent obesity and eating disorders. Exposure to images of bodies of different sizes has been suggested to change the perception of ‘normal’ body size in others. We tested whether exposure to different-sized (otherwise identical) bodies changes perception of own and others' body size, satisfaction with body size and amount of chocolate consumed. In Study 1, 90 18–25-year-old women with normal BMI were randomized into one of three groups to complete a 15 min two-back task using photographs of women either of ‘normal weight’ (Body Mass Index (BMI) 22–23 kg m^−2^), or altered to appear either under- or over-weight. Study 2 was identical except the 96 participants had high baseline body dissatisfaction and were followed up after 24 h. We also conducted a mega-analysis combining both studies. Participants rated size of others' bodies, own size, and satisfaction with size pre- and post-task. Post-task ratings were compared between groups, adjusting for pre-task ratings. Participants exposed to over- or normal-weight images subsequently perceived others' bodies as smaller, in comparison to those shown underweight bodies (*p* < 0.001). They also perceived their own bodies as smaller (Study 1, *p* = 0.073; Study 2, *p* = 0.018; mega-analysis, *p* = 0.001), and felt more satisfied with their size (Study 1, *p* = 0.046; Study 2, *p* = 0.004; mega-analysis, *p* = 0.006). There were no differences in chocolate consumption. This study suggests that a move towards using images of women with a BMI in the healthy range in the media may help to reduce body dissatisfaction, and the associated risk of eating disorders.

## 1. Background

Body dissatisfaction is a risk factor for eating disorders [1], low mood, obesity and weight gain in adolescents and young adults of both genders [2–5]. It is common, with prevalence studies finding 35% of adolescent girls [6], and two-thirds of adult women [7], are dissatisfied with their bodies. One main component of body dissatisfaction relates to body size, with over 75% of adolescent girls [8], and over 95% of adult women [9] reporting a wish to be thinner, and 74% of adult women reporting a wish to lose weight [10]. This is often ascribed to ‘internalization of the thin ideal’ (the idea that individuals, to differing amounts, try to conform to media-driven, socially defined ideas of attractiveness, including body size) [11,12]. If it were possible to alter perception of own body size, this could potentially be used as a way of reducing body dissatisfaction and its negative consequences, including obesity and weight gain [5], in the general population.

There is some evidence that exposing people to images of bodies of different sizes alters their perception of *others*' body size, in that it changes their idea of what is considered to be a ‘normal’ size for others' bodies. The female body size viewed as being most ‘normal’ becomes thinner after exposure to thinner bodies, and larger following exposure to larger bodies [13,14]. Viewing photographs of males whose body mass index (BMI) is in the obese, as opposed to normal, category leads to both male and female participants judging a man with a BMI in the overweight range to have a healthy, normal weight [15,16]. Although two of these studies included only images of women, and two only images of men, the results across both studies were consistent in terms of a change in perception of what constitutes a ‘normal’ body size in others. The body size perceived as most attractive also became smaller following exposure to thinner as opposed to larger bodies [13,14].

However, studies looking at the impact of exposure to bodies of different sizes on perception of *own* body size have been small (*N*s of 14 and 16) [17,18]. Participants in these two studies were shown ‘thin’ or ‘fat’ modified photographs of self or others, and were then asked to pick the most accurate photograph of themselves: those shown thinner-than-actual photographs of self or others judged a thinner-than-actual picture of self as most accurate; those shown fatter-than-actual photographs of self or others subsequently judged a fatter-than-actual photograph as most accurate. This suggests that photographs of others may change perception of own size, but the studies were too small to be confident that these results did not arise by chance.

In addition to whether exposure to bodies of different sizes changes perception of own body *size*, another important question is whether it changes *satisfaction* with own body size. Meta-analysis [19] concludes that in general women feel less satisfied with their bodies after viewing photographs of thin models compared to neutral images or normal-sized models. However, of the 80 studies included in this meta-analysis, 75 either had no control group, or compared viewing thin models with neutral images of some sort, including two which used images of older men and women, and infants and children playing [20,21], which does not give insight into what aspects of the models' bodies might be having this effect. Of those using women's bodies in the control group, one study used images which were chosen to vary according to attractiveness rather than weight [22], and one used television footage of ‘thin and attractive’ women in the experimental group and compared this with ‘not thin or attractive’ women in the control group [23], again leaving it uncertain whether the differences were due to factors relating to attractiveness or to weight. Two studies used clothing adverts or fashion models from women's magazines in the experimental group, but compared them with ‘pictures from specialized women's clothing catalogues’ for ‘more amply furnished women’ in one case [24], and ‘overweight bordering on obese women’ from other magazines in the other [25]. It seems likely that such images would vary in terms of the attractiveness and glamour of the images as well as their size and weight. One study attempted to look at the effects of body satisfaction of photographs which varied on weight alone, using the face of one woman pasted onto either a relatively slim or an overweight body [26]. This study found lower levels of body satisfaction in the group shown a single overweight image. However, the study did not measure baseline levels of body satisfaction, so it is possible that these differences between groups were pre-existing. Interpreting these results is further complicated by the study scenario, in which the women were told that they were about to meet a male participant who would choose whether he would rather go on a date with them, or the other woman, whose photograph they were purportedly being shown. One further small study (*N* = 40, randomized into four groups) used a single black and white print advert for Kraft cheese, featuring an ‘attractive female model’ in two conditions, and a version using the model altered to appear overweight using Adobe Photoshop (no further details of the manipulation are reported) in a further two conditions; participants rated self-attractiveness after viewing the image for 15 s. Self-attractiveness ratings were lower following exposure to the ‘overweight’ models, but again no baseline measurements were taken [27].

One recent paper has examined whether giving feedback on participants' judgement as to the size of others' bodies can reduce clinical eating disorder symptom scores. Forty participants with high baseline body dissatisfaction were shown a series of CGI images of women's bodies and asked to rate each of them as ‘fat’ or ‘thin’. They were then randomized to either complete a ‘training’ task in which they were given feedback regarding whether their response was correct or not, with the aim of shifting the threshold where they perceived a body as fat, or a control task where the feedback was consistent with their baseline judgements. The training task successfully shifted their perception of the cut-off between ‘fat’ and ‘thin’ bodies within the task, and resulted in decreased levels of eating disorder symptoms (shape, weight and eating concerns), which were sustained for two weeks [28]. Results were replicated in 20 patients with atypical anorexia nervosa, raising the possibility that such interventions may be a useful adjunct in eating disorder treatment.

To our knowledge, no studies to date have investigated the effect of shifting perception of body size on any behavioural outcome, such as food consumption. Whether food consumption increases or decreases might enhance our understanding of how body dissatisfaction can influence subsequent eating disorders or weight gain.

**Figure 1.**
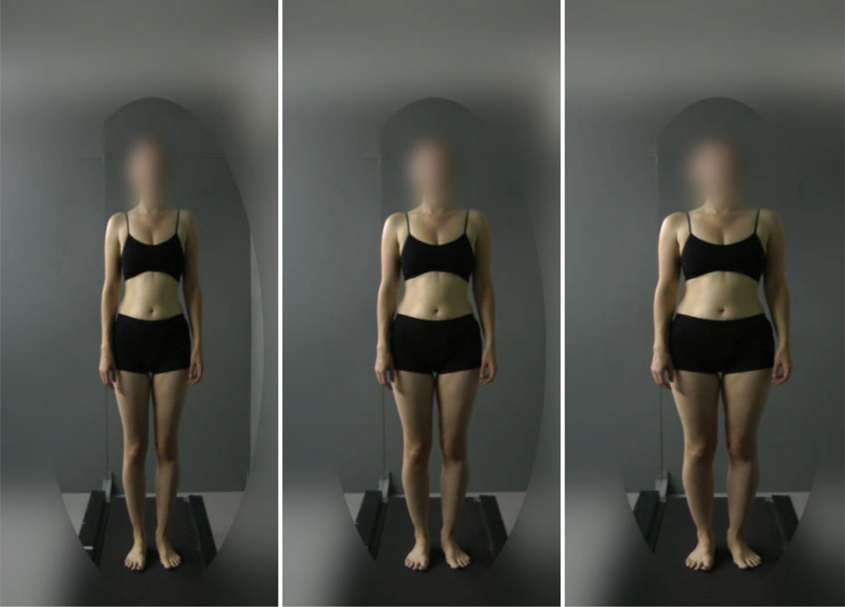
Example of stimuli used (left: ‘underweight’; middle: ‘normal weight’; right: ‘overweight’).

The studies presented here build on the previous literature to investigate whether the change in perception of body size of others extends to perception of and satisfaction with own body size, even without giving explicit ‘training’ instructions about which bodies are ‘fat’ or ‘thin’, and how long such effects last. We used a tightly controlled set of images, comprising photographs of the same women, whose BMI was in the middle of the normal range (22–23 kg m^−2^) (‘normal weight’), either unmodified, or modified to appear either ‘overweight’ or ‘underweight’ (figure 1). Participants were randomized to see one weight category of images. After seeking to confirm the previous findings that exposure to such images changes perception of the weight of others' bodies, we went on to test, in a sample six times larger than that previously used, the hypothesis that such exposure changes subsequent perception of the size of the participant's own body. We then tested whether such exposure to different sized bodies can change satisfaction with own body size. We also investigated whether changes in perception of or satisfaction with size resulted in behavioural differences in terms of amount of chocolate consumed. Study 2 aimed to replicate the results from Study 1 in a sample of participants with high baseline levels of body dissatisfaction, which may be considered to be more similar to an eating disorder patient group, and also investigated whether the effects would persist for 24 h. Given the higher prevalence of body dissatisfaction in women than men, we decided that these studies would focus on women only.

In both studies, we hypothesized that following exposure to images of ‘underweight’, as opposed to ‘normal’ or ‘overweight’ bodies, participants would (1) judge others' bodies as larger, (2) judge their own bodies as larger, and (3) be less satisfied with their body size. We further hypothesized that increase in satisfaction with body size would lead to (4) increased chocolate consumption following the task, possibly due to decreased worry about own weight. In Study 2, we further hypothesized (5) that changes would persist to the following day.

## 2. Methods: Study 1

### 2.1. Participants

We recruited female 18–25 year olds from the general population, by email invitation to a database of volunteers willing to be contacted about psychology research at the University of Bristol (consisting of University of Bristol students), and through advertisements on the University of Bristol Precinct. Potential participants completed a brief electronic screening questionnaire giving their age, sex, fluency in English, height, weight, and whether they had a previous diagnosis of an eating disorder; they were not asked for details of their ethnicity or socio-economic status.

Participants were eligible to take part if they were female, English (or fluent in English), had no history of a diagnosed eating disorder, and had a body mass index (BMI) of 19–25 kg m^−2^ (normal range). Participants were offered £5 of ‘Love to Shop’ vouchers for their time.

### 2.2. Measures

Participants completed the Body Dissatisfaction (BD) subscale of the Eating Disorder Inventory (EDI) [29] online before attending the session. The Body Dissatisfaction subscale comprises nine items, and we scored them as suggested for a non-clinical population [30]: four from 1 (Never) to 6 (Always) (e.g. ‘I think that my thighs are too large’), and five from 1 (Always) to 6 (Never) (e.g. ‘I think that my hips are just the right size’). The Body Dissatisfaction subscale of the EDI has an internal reliability of 0.90 in a clinical, and 0.94 in a non-clinical, sample [30]. Participants completed the PHQ-9 (Patient Health Questionnaire) [31] during the session, before completing the task, as mood may impact on body dissatisfaction [32,33]. The PHQ-9 is a 9 item depression measure consisting of questions which score the DSM-IV depression criteria as ‘0’ (not at all) to ‘3’ (nearly every day). The scale has an internal reliability of 0.89. Before and after completing the task, participants estimated the size of four normal-weight stimuli on the computer screen, on a Visual Analogue Scale (VAS) from slightly underweight (1) to slightly overweight (7). Two of these stimuli were from the same set of images as the task stimuli (although not images which had been used in the tasks), and were dressed like the task stimuli in a sports bra and shorts, and two were fully clothed. Participants also completed VAS of own size, from 0 (too thin) to 10 (too fat), and satisfaction with own size, from 0 (not at all satisfied) to 10 (extremely satisfied), both before and after the task.

### 2.3. Stimuli

Stimuli used for the task were created from full body photographs of 10 different women aged between 18 and 25 years with a BMI of 22 to 23 kg m^−2^ and of White ethnicity, selected from a set of images of women with known BMI held in the School of Experimental Psychology, University of Bristol. These stimuli were used for the control group, ‘normal weight’. The stimuli were modified by increasing the width of the images of women's bodies (initially 880 pixels) by 150 pixels to create an ‘overweight’ group, and decreasing the width of the women's bodies by 150 pixels to create an ‘underweight’ group. Images were cropped so the width of all photographs remained 880 pixels. Faces were blurred to preserve the privacy of the women in the photographs, and to prevent subjects using faces to recognize the stimuli. An example of the stimuli used is given in figure 1 in black and white; full colour images were used in the task.

### 2.4. Procedure

Participants attended the University of Bristol, dressed in close-fitting clothing, as requested in the Participant Information Sheet and in the email inviting them. They provided informed consent, consenting to the tasks they would undertake, but remaining blind to the hypothesis and purpose of the study. They were tested individually, by one of two experimenters (H.B. and R.C.). They were randomized to one of the three groups by a computer-generated random sequence to ensure balance across groups. An author (K.S.B.) not involved in any testing or participant contact assigned letters to the three training conditions; experimenters and participants were blind to which training condition was which.

Participants completed the pre-task measures described above. They then completed a single session of the training. Following this, they were asked to remove outer layers of clothing (e.g. cardigans, jumpers, jackets, coats), look at themselves in a full length mirror, and then complete the post-task measures. The purpose of using the mirror was to ensure that the participant saw their own body in relation to the bodies to which they had been exposed. The experimenter then weighed them and measured their height without shoes. They were then asked to take a seat outside the room, the experimenter explaining ‘I need to check the computer has registered the tasks correctly’, and they were offered a bowl containing 300 g chocolate (Maltesers) while they waited. Maltesers were chosen as they are a palatable snack food that most people like, and because since they are quite small, people might choose to eat a variable quantity. After 2–3 min, they were invited back into the room, the remaining chocolate was weighed and they were debriefed.

The training session consisted of a two-back working memory task. The stimuli (photographs of the 10 women adjusted to be either ‘over’, ‘under’ or ‘normal’ (BMI 22–23 kg m^−2^) weight) were presented sequentially (and repeatedly) to participants, who were instructed to respond as to whether the image was a ‘target’ (i.e. had the same identity as the individual presented two trials previously) or a ‘non-target’ (i.e. had a different identity from the individual presented two trials previously). This was to ensure that participants paid attention to the images displayed, which has been shown to enhance training effects [34]. In the ‘underweight’ condition, the 10 images modified to appear ‘underweight’ were displayed. In the overweight condition, the 10 images modified to appear ‘overweight’ were displayed. In the control condition, the 10 images of ‘normal weight’ women were displayed.

The tasks were programmed using E-Prime version 2.0 software (Psychology Software Tools Inc., Pittsburgh, PA, USA). Each image was displayed for 2000 ms, with a 20 ms inter-trial interval. To prevent low-level visual adaptation to the images, they were spatially jittered by an amount varying randomly from the centre of the screen by up to ±51 pixels in the x coordinate and ±38 pixels in the y coordinate (approximately 10% in each direction of the 1024 by 768 pixel display). Image size was 562 by 765 pixels, with a viewing distance of approximately 1 m. Failure to respond within the 2000 ms presentation resulted in no response being recorded for that trial. Each task block consisted of 48 trials, in which 8 ‘targets’ were presented. A total of 7 blocks, giving a total of 336 trials, were presented, taking approximately 15 min for each participant.

### 2.5. Statistical analysis

Data were checked to ensure that no data were missing. The primary outcome was judgement of own body size (VAS from too thin (0) to too fat (10)) post-task. The secondary outcome was participant satisfaction with their body size after the intervention (VAS from not at all satisfied (0) to extremely satisfied (10)). Other outcome measures included perception of size of the women with BMI 22–23 kg m^−2^ shown after the task, and weight of chocolate taken by participants after finishing the task.

For each outcome measure, we performed a linear regression analysis, treating randomization group as the categorical exposure variable and adjusting for the participants' baseline on each measure, by including in the regression analysis. All analyses were repeated adding adjustments for age, BMI and PHQ-9 score. The data that form the basis of the results presented here are available with restricted access from the data.bris Research Data Repository (https://data.bris.ac.uk/data) doi:10.5523/bris.rydgtj6qorrf1ndeh2e3c74g8, with author permission.

Scores for individual measurements that were more than three standard deviations from the mean were judged to be outliers, and sensitivity analyses were repeated excluding such outliers.

Power calculation shows that three groups of 30 gave us 90% power at a 5% alpha level to detect an effect size of *d* = 0.38.

## 3. Results: Study 1

### 3.1. Descriptive data

**Figure 2.**
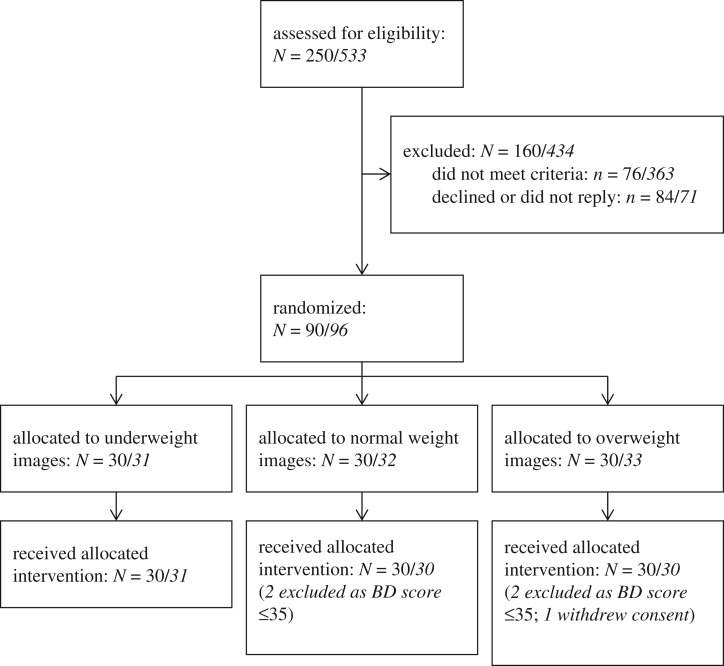
CONSORT diagram for Studies 1 and 2 *(Study 2 italicized)*.

We recruited 90 women between 11th November 2012 and 16th December 2013. Study recruitment is shown in the CONSORT diagram (figure 2) and descriptive data in table 1.

**Table 1.** Descriptive data on groups at baseline and post-training.

	Study 1				Study 2			
	all groups mean (s.d.)	adapted to ‘underweight’ mean (s.d.)	adapted to ‘normal weight’ mean (s.d.)	adapted to ‘overweight’ mean (s.d.)	all groups mean (s.d.)	adapted to ‘underweight’ mean (s.d.)	adapted to ‘normal weight’ mean (s.d.)	adapted to ‘overweight’ mean (s.d.)
age	20.4 (1.86)	20.6 (1.94)	20.1 (1.55)	20.3 (2.09)	21.4 (2.03)	21.5 (2.08)	21.2 (2.16)	21.5 (1.89)
body dissatisfaction score (0–54)	30.9 (7.2)	30.7 (5.44)	32.3 (7.71)	29.8 (8.20)	39.4 (3.89)	40.0 (4.94)	39.1 (3.06)	39.1 (3.43)
measured height (m)	1.66 (0.06)	1.66 (0.06)	1.66 (0.07)	1.65 (0.05)	1.66 (0.07)	1.65 (0.08)	1.68 (0.07)	1.65 (0.06)
measured weight (kg)	60.0 (6.52)	61.2 (4.99)	59.6 (7.72)	59.1 (6.60)	60.5 (7.04)	60.9 (8.26)	61.7 (7.77)	60.1 (6.47)
measured BMI (kg m^−2^)	21.8 (1.77)	22.3 (1.60)	21.5 (1.77)	21.7 (1.89)	22.1 (1.85)	22.3 (1.98)	21.9 (1.84)	22.1 (1.77)
PHQ-9 score	3.8 (3.05)	3.4 (2.39)	5.0 (3.60)	2.9 (2.73)	6.5 (4.17)	7.65 (4.83)	6.47 (4.31)	5.3 (2.90)
baseline rating of size of computer images (1–7)	4.78 (0.84)	4.80 (0.72)	4.86 (0.89)	4.68 (0.90)	5.09 (0.99)	4.94 (0.91)	5.19 (1.04)	5.14 (1.05)
post-training rating of size of computer images (1–7)	4.54 (0.93)	5.03 (0.84)	4.48 (0.82)	4.13 (0.92)	4.57 (0.91)	4.85 (0.77)	4.69 (0.64)	4.16 (1.12)
day 2 rating of size of computer images (1–7)	—	—	—	—	4.83 (0.77)	5.02 (0.75)	5.0 (0.63)	4.52 (0.82)
					(*N* = 84)	(*N* = 28)	(*N* = 26)	(*N* = 30)
baseline rating of own size (0–10)	5.99 (1.01)	5.87 (0.96)	6.12 (0.97)	5.99 (1.13)	6.73 (1.01)	7.01 (0.96)	6.85 (1.02)	6.33 (0.96)
post-training rating of own size (0–10)	5.74 (1.07)	5.88 (1.09)	5.79 (0.97)	5.53 (1.15)	6.26 (1.20)	6.82 (1.03)	6.27 (1.22)	5.68 (1.09)
day 2 rating of own size (0–10)	—	—	—	—	6.58 (1.04)	6.89 (0.97)	6.73 (1.10)	6.15 (0.91)
					(*N* = 84)	(*N* = 28)	(*N* = 26)	(*N* = 30)
baseline satisfaction with own size (0–10)	5.28 (2.06)	5.60 (2.06)	4.77 (1.64)	5.48 (2.37)	3.92 (1.68)	3.72 (1.87)	3.85 (1.62)	4.20 (1.56)
post-training satisfaction with own size (0–10)	5.79 (1.96)	5.77 (2.12)	5.65 (1.61)	5.96 (2.14)	4.45 (1.71)	3.81 (1.66)	4.32 (1.55)	5.25 (1.63)
day 2 satisfaction with own size	—	—	—	—	3.99 (1.58)	3.54 (1.53)	3.73 (1.50)	4.63 (1.54)
					(*N* = 84)	(*N* = 28)	(*N* = 26)	(*N* = 30)
chocolate consumed post-training(g)	7.71 (6.16)	6.10 (5.6)	8.30 (6.10)	8.73 (6.64)	6.00 (7.57)	3.9 (4.85)	8.48 (9.15)	5.86 (7.82)

### 3.2. Perception of size of computer images (Hypothesis 1)

**Figure 3.**
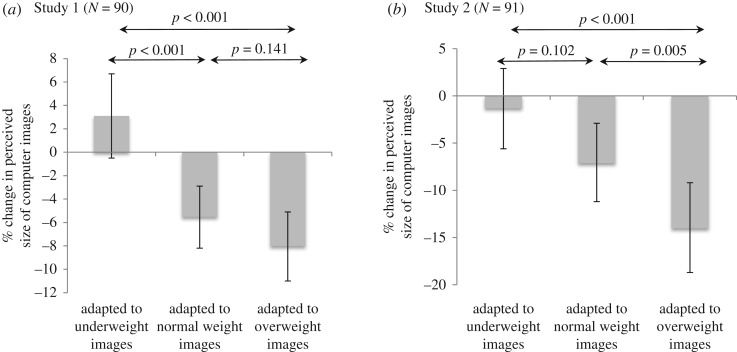
Mean percentage perceived change in size of computer images, pre- to post-task, by group (error bars represent 95% confidence intervals).

There was evidence of a difference between the means for the three groups in their judgement of the size of the computer images shown following exposure to different sized images, with those exposed to ‘overweight’ images during the task perceiving subsequent images as smaller than those exposed to normal or ‘underweight’ images (*p* < 0.001) (figure 3*a*). Adjustment for potential confounders (baseline PHQ-9, age, BMI) did not alter these results substantially. Full adjusted and unadjusted results for all measures are given in a supplementary online table (electronic supplementary material, table S2).

### 3.3. Perception of own body size (Hypothesis 2)

**Figure 4.**
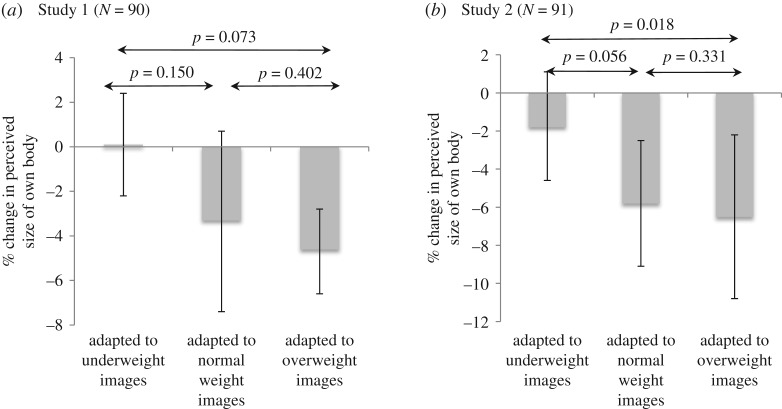
Mean percentage perceived change in own body size, pre- to post-task, by group (error bars represent 95% confidence intervals).

We did not find evidence to support a difference between the three groups in their mean judgement of their own size following exposure to different sized images (*p* = 0.073) (figure 4*a*). Adjusting for potential confounders did not alter these results substantially.

### 3.4. Satisfaction with own body size (Hypothesis 3)

**Figure 5.**
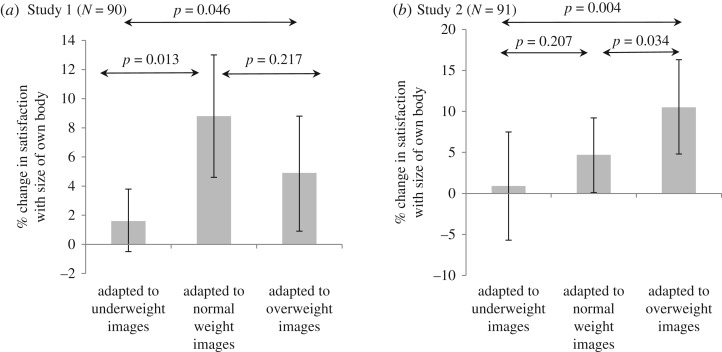
Mean percentage change in satisfaction with own body size, pre- to post-task by group (positive numbers indicate higher levels of satisfaction following task) (error bars represent 95% confidence intervals).

There was evidence for group differences in mean satisfaction with own size following exposure to different sized images (*p* = 0.046), with those exposed to ‘underweight’ images being less satisfied than those exposed to ‘normal’ or ‘overweight’ images (figure 5*a*). Adjusting for potential confounders made no substantial difference to the results.

### 3.5. Chocolate consumption (Hypothesis 4)

**Figure 6.**
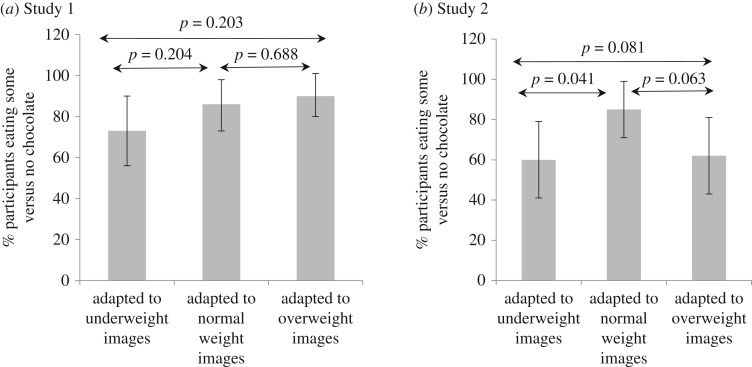
Percentage of participants eating some versus no chocolate following the task (error bars represent 95% confidence intervals).

The weight of chocolate consumed was not normally distributed, so a binary variable was created of eating ‘no’ versus ‘any’ chocolate. Treating the three experimental groups as a categorical variable, logistic regression found no differences in chocolate consumption between groups (*p* = 0.203) (figure 6*a*).

### 3.6. Sensitivity analysis excluding outliers

Removing outliers resulted in excluding four participants with scores for individual measurements that were more than three standard deviations from the mean: one participant who reported her own size at baseline as 10 on the 10-point VAS scale, one who reported a change in her perception of her own size of 4 points on the 10-point VAS scale, and two who reported an increase in satisfaction of 3.5 and 3.85 respectively on the 10-point VAS scale. Analyses were repeated as described above, but excluding these outliers (electronic supplementary material, table S3).

There were no differences in results for judgement of the size of the computer images before and after the task. In judgement of own size, the statistical evidence for a difference between groups strengthened considerably (*p* = 0.004). There were no major differences in satisfaction with own size following removal of outliers. Adjusting for confounding variables did not substantially alter any results.

## 4. Methods: Study 2

Study 2 replicated Study 1 in a group with high baseline body dissatisfaction, in order to see whether results could be replicated in a group with more relevance to clinical populations. Differences in methodology were slight and are outlined below. The study protocol was preregistered on the Open Science Framework (https://osf.io/sngb6/).

### 4.1. Participants

Inclusion criteria required a baseline body dissatisfaction score of greater than 35 on the Body Dissatisfaction subscale of the Eating Disorder Inventory [29]; this value was chosen as it corresponded to the top quartile of scores in Study 1. Criteria were also widened to include those with a reported body mass index (BMI) of 18 to 25 kg m^−2^ as we anticipated it being harder to recruit sufficient numbers with a high BD score, and in Study 1 we had to exclude many participants with a BMI between 18 and 19. Reimbursement was increased to £10, due to the requirement to attend follow-up the next day.

### 4.2. Measures and stimuli

Measures were identical to Study 1; participants completed the same measures at 24 h follow-up. Stimuli were identical to Study 1.

### 4.3. Procedure

The experiment was conducted by four authors (E.L., E.B., M.S. and H.A.). Day 1 study procedure was identical, except that participants were not debriefed. Participants attended 24 h follow-up. They looked at themselves in a full-length mirror, then completed the same measures as previously. Finally, they repeated the computer task using the ‘normal’ weight women, in order to reverse any increase in body dissatisfaction caused by exposure to ‘underweight’ women. They were then debriefed.

### 4.4. Statistical analysis

Outcomes and analysis were identical to Study 1, with additional analysis of results from 24 h follow-up, to investigate whether between-group differences in perception of computer images, and perception of and satisfaction with own size were present after 24 h. The data forming the basis of the results presented here are available open access from the data.bris Research Data Repository (https://data.bris.ac.uk/) doi:10.5523/bris.o1pqic3gep9n12c5mh0q0ojwj. A power calculation showed that three groups of 31 participants gives 90% power to detect between randomization group differences of 0.5 on a 10-point scale at a significance level of 0.05.

We also subsequently conducted a series of mega-analyses, in which we repeated all the analyses we have described, incorporating the results from both Study 1 and Study 2 into each analysis. The analyses were identical to those described for the individual studies, except that we included a variable to indicate whether each data point was from Study 1 or Study 2.

## 5. Results: Study 2

### 5.1. Descriptive data

We recruited 96 women between 29 July 2014 and 9 August 2015 (see figure 2 for CONSORT diagram). Four participants were excluded as they had been erroneously recruited with Body Dissatisfaction scores ≤35, despite inclusion criteria requiring a score greater than 35. One participant withdrew her consent following participation. Table 1 shows descriptive data.

### 5.2. Perception of size of computer images (Hypothesis 1)

There was evidence of a difference between the means for the three groups in their judgement of the size of the images shown following exposure to different sized images, consistent with the previous study, with those exposed to ‘overweight’ images during the task viewing subsequent images as smaller than those shown normal or ‘underweight’ images (*p* < 0.001) (figure 3*b*; electronic supplementary material, table S2). Adjusting for potential confounders (baseline PHQ-9, age, BMI) did not alter these results substantially (electronic supplementary material, table S2).

### 5.3. Perception of own body size (Hypothesis 2)

There was evidence of a difference between the means for the three groups in their judgement of their own size following exposure to different sized images, with those shown ‘overweight’ images subsequently judging their own body as smaller than those shown ‘normal’ or ‘underweight’ images (*p* = 0.018) (figure 4*b* and electronic supplementary material, table S2). Adjusting for potential confounders did not alter these results substantially.

### 5.4. Satisfaction with own body size (Hypothesis 3)

There was evidence for group differences in mean satisfaction with own size following exposure to different sized images, with those shown overweight images subsequently being more satisfied with their own bodies than those shown ‘underweight’ or ‘normal weight’ images (*p* = 0.004) (figure 5*b* and electronic supplementary material, table S2). Adjusting for potential confounders made no substantial difference to the results.

### 5.5. Chocolate consumption (Hypothesis 4)

The weight of chocolate consumed was not normally distributed, so a binary variable was created of eating ‘no’ chocolate versus eating ‘any’ chocolate. Treating the three groups as a categorical variable, logistic regression analysis found weak evidence for between-group differences in chocolate consumption, with those in the ‘underweight’ and ‘overweight’ groups both eating less chocolate than those in the ‘normal weight’ group (*p* = 0.081) (figure 6*b*).

### 5.6. Twenty-four hour follow-up (Hypothesis 5)

There was some loss to follow-up: 84 of the 91 participants attended follow-up (92%), of whom 26 were in the group shown ‘normal weight’ images, 28 in the group shown ‘underweight’ images and 30 in the group shown ‘overweight’ images. Analysing using only data from those participants who attended on both days, we found that between-group differences in perceived size of computer images persisted at 24 h follow-up (*p* < 0.001), with differences in the same direction as in the post-task follow-up (electronic supplementary material, table S2). Results persisted following adjustment for confounding variables. There was no evidence that changes in perception of own size persisted to follow-up (*p* = 0.311). Differences in satisfaction with own size did persist (*p* = 0.032), although the strength of the evidence for this difference reduced following adjustment for confounders (*p* = 0.080). Repeating analyses including all participants, carrying forward the last available observation (i.e. those collected immediately after the task) in those who did not attend follow-up, did not substantially alter these results.

### 5.7. Sensitivity analysis excluding outliers

Removing outliers resulted in excluding five participants with scores for individual measurements that were more than three standard deviations from the mean (one scoring 10 and one scoring 3 for pre-test own size, two with change in size scores of −3.9 and +2 and one with a mean post-task score of the computer images of 1). Analyses were repeated excluding these outliers. There was no difference in results for judgement of the size of the computer images before and after the task. In judgement of own size, statistical evidence for a difference between groups strengthened considerably (*p* = 0.005) (electronic supplementary material, table S3); the difference in perception of own size following exposure to ‘overweight’ versus ‘normal weight’ images remained stable. Adjusting for confounding variables did not substantially alter any of these results.

### 5.8. Mega-analysis of Studies 1 and 2

Combining the results from Studies 1 and 2 strengthened the findings throughout, and we found strong statistical evidence in support of hypotheses 1 to 3 (electronic supplementary material, table S2). Adjusting for confounders, and repeating the analyses excluding outliers (as defined separately for Study 1 and Study 2) did not change these findings.

## 6. Discussion

The aims of the study were to test in a sample six times larger than that previously used whether exposure to bodies of different sizes would extend beyond changing perception of others' bodies to also change perception of own body size. We found that this was the case in Study 2 (participants with high baseline levels of body dissatisfaction), and in mega-analyses of Studies 1 and 2. Furthermore, we tested the hypothesis that these changes in perception of own size would also change satisfaction with own size, and this was demonstrated and replicated across the two studies. Finally, we found that changes in satisfaction with own body size lasted until 24 h follow-up.

We confirmed findings from previous studies [13,14,17,18,28] that exposing women to images of women's bodies of different sizes alters their perception of the size of subsequent bodies, with exposure to larger bodies leading to subsequent perception of other bodies as smaller. We confirmed that this is found in those with, and without, high baseline levels of body dissatisfaction. In Study 2, and in the mega-analysis, we found good evidence that exposing participants to images of ‘overweight’ as opposed to ‘underweight’ or ‘normal weight’ bodies leads to them perceiving their own size as smaller. We also showed in both studies that satisfaction with own size changes following exposure to images of women's bodies of different sizes, with exposure to larger bodies resulting in increased satisfaction. This finding was stronger in the group of participants who were dissatisfied with their bodies at baseline. Differences in satisfaction with own body size, and perception of body size of others persisted to 24 h follow-up, although differences in perception of own size did not persist. We did not find any overall effect of exposure to images of different sizes on chocolate consumption, which may be due to individual variation in whether a change in satisfaction with own size leads to the consumption of more or less chocolate.

Studying the differences between groups more closely, changes in perception of own and others' size appears to be predominantly driven by decreases in perception of own size following exposure to normal or overweight images. Exposure to ‘underweight’ images had less effect on subsequent perception of own and others' size. This may be because our ‘underweight’ images are in fact very similar in size to the media images surrounding us, and may be larger than average media images. For example, a study of Internet models found that over 50% had a BMI of ≤18 [35]. It is likely that body size norms [36] are strongly affected by previous exposure to media images, so further exposure to ‘underweight’ images may make little difference.

The current studies build on previous research in demonstrating in a larger sample than that previously used, that exposure to bodies of different sizes can alter perception of and satisfaction with own body, and this occurs in all women and to a greater extent in those with high baseline body dissatisfaction. Experimenters and participants were blind to which training study was which, and the study was tightly controlled, in that all groups saw images of the same women, manipulated to appear in different sizes. The second study replicated the methods of the first, and was preregistered with defined hypotheses and primary outcome measures.

However, there were some limitations to the study. First, participants were almost all students at the University of Bristol, and we do not have data on their ethnicity or socio-economic status; they therefore may not be representative of the national population of 18–25 year olds. Second, the images created were not very sophisticated, merely varying the overall width of the bodies rather than altering their proportions in any other way; we did not pilot the images to see whether they were viewed as realistic, and they were all of women of White ethnicity. However, the images are more sophisticated than those used in some previous studies in that they are photographs of real women, rather than 2D or 3D sketches. Third, our ‘normal’ images comprised images of women with a known BMI of 22 to 23 kg m^−2^, but we do not know the BMI of the groups we created by altering the widths of the images. The mean BMI of the participants (21.8 kg m^−2^ in Study 1; 22.1 kg m^−2^ in Study 2) was at the lower end of the range of the BMI of the ‘normal’ weight group (22 to 23 kg m^−2^) whose images we used. Fourth, at baseline, participants rated the images with a BMI of 22 to 23 kg m^−2^ as being towards the overweight end of the scale (mean of 4.78 on a 7-point scale (Study 1); 5.09 (Study 2)), despite this BMI being in the middle of the healthy range of BMI. Therefore, those completing the ‘normal’ weight version of the task may have been exposed to images they considered to be slightly overweight. This does appear to be the case: exposure to ‘normal’ weight images led to viewing subsequent computer images as smaller, perceiving own size as smaller and being more satisfied with own size. Fifth, the wording of the task in which participants judged their own size included the phrases ‘too fat’ and ‘too thin’, which may have drawn on their feelings of satisfaction with their body as well as their perception of their actual size. Sixth, participants may have guessed the purpose of the study, and results may have been affected by demand characteristics; we did not check participant awareness of the study hypothesis. Seventh, the follow-up time was only 24 h, and we therefore do not know whether effects may last longer than this. Eighth, the choice of chocolate as a snack may not have been ideal, both because it is a food that some people crave, and because the craving often involves both approach and avoidance, as well as feelings of guilt [37].

Our findings are potentially important in view of the current media environment, where women are constantly exposed to images of underweight women. We have demonstrated here that relatively short exposure to images of normal women's bodies of different sizes can change perception of the size of both own and others' bodies, and satisfaction with own size, with particularly marked effect in those who feel more negative about their bodies at baseline. It is possible that the change in satisfaction with own size may be due to exposure to different-sized bodies changing the internalized ‘ideal’ body size to which women compare their own bodies, as well as to a change in perceived own size. In either case, the results of our study suggest that encouraging media outlets to choose models of a normal body size would be likely to lead to women perceiving their own size as smaller and feeling more positive about their own body size: this may in turn reduce levels of body dissatisfaction, a known risk factor for eating disorders and obesity. Some evidence suggests that choosing larger models would not diminish the effectiveness of advertising [38].

The effect of presenting images of larger women in changing perception of own body size, and increasing body size satisfaction, particularly in highly body-dissatisfied women, also suggests that such an intervention may have a role to play in the prevention of eating disorders and obesity. Intervening to reduce high levels of body dissatisfaction may be a useful adjunct in, for example, eating disorder prevention programmes.

Future research could usefully investigate whether such an intervention is effective in patients with a diagnosed eating disorder. In such patients, perceiving own body size as larger than it is in reality is a core part of the disorder [39], which is resistant to current treatments [40] and is predictive of relapse [1].

## 7. Conclusion

In conclusion, our results put forward a strong public health message that increasing the number of normal and larger women in the media may reduce levels of body dissatisfaction in the population, and thus would have the potential to reduce rates of weight gain, obesity and eating disorders.

## Supplementary Material

Table 2

## Supplementary Material

Table 3

## Data accessibility

The data forming the basis of the Study 1 results presented here are available from the data.bris Research Data Repository (https://data.bris.ac.uk/data) doi:10.5523/bris.rydgtj6qorrf1ndeh2e3c74g8, with author permission; results from Study 2 are available open access from the data.bris Research Data Repository (https://data.bris.ac.uk/) doi:10.5523/bris.o1pqic3gep9n12c5mh0q0ojwj.
