# Supplementary material for: Effects of exposure to bodies of different sizes on perception of and satisfaction with own body size: two randomized studies
Source: R Soc Open Sci. 2018 May 9;5(5):171387. doi: 10.1098/rsos.171387 (PMC5990741; doi:10.1098/rsos.171387)
Supplement: Table 2 [file rsos171387supp1.docx]

Table 2: Perception of size of others, own size and satisfaction with own size (Studies 1 & 2)

|  |  | Comparison with those adapted to normal weight images* | | | Comparison with those adapted to normal weight images, adjusted for age, BMI and PHQ-9 score* | | |
| --- | --- | --- | --- | --- | --- | --- | --- |
|  |  | Adapted to underweight images | Adapted to overweight images | Test of Trend across groups | Adapted to underweight images | Adapted to overweight images | Test of Trend across all groups |
| Perceived size of computer images (post-task rating adjusted for pre-task rating) | Study 1 | 0.59 (0.30, 0.87), p<0.001 | -0.21 (-0.50, 0.07), p=0.141 | p<0.001 | 0.66 (0.37, 0.96), p<0.001 | -0.17 (-0.46, 0.13), p=0.269 | P<0.001 |
|  | Study 2 | 0.29 (-0.06, 0.64), p=0.102 | -0.51 (-0.86, -0.15), p=0.005 | p=0.001 | 0.33 (-0.03, 0.68), p=0.069 | -0.48 (-0.84, -0.13), p=0.008 | P<0.001 |
|  | Studies 1&2 combined | 0.44 (0.22, 0.68), p<0.001 | -0.37 (-0.60, -0.14), p=0.002 | p<0.001 | 0.50 (0.27, 0.72), p<0.001 | -0.34 (-0.58, -0.12), p=0.003 | p<0.001 |
| Perceived own size (post-task rating adjusted for pre-task rating) | Study 1 | 0.28 (-0.10, 0.66), p=0.150 | -0.16 (-0.54, 0.22), p=0.402 | p=0.073 | 0.28 (-0.13, 0.68), p=0.180 | -0.12 (-0.53, 0.28), p=0.546 | p=0.132 |
|  | Study 2 | 0.44 (-0.01, 0.90), p=0.056 | -0.23 (-0.70, 0.24), p=0.331 | p=0.018 | 0.40 (-0.06, 0.85), p=0.085 | -0.30 (-0.77, 0.17), p=0.215 | p=0.018 |
|  | Studies 1&2 combined | 0.36 (0.06, 0.65), p=0.018 | -0.19 (-0.48, 0.11), p=0.217 | p=0.001 | 0.31 (0.01, 0.60), p=0.04 | -0.20 (-0.50, 0.10), p=0.197 | p=0.004 |
| Satisfaction with own size (post-task rating adjusted for pre-task rating) | Study 1 | -0.60 (-1.07, -0.13), p=0.013 | -0.29 (-0.77, 0.18), p=0.217 | p=0.046 | -0.67 (-1.16, -0.18), p=0.008 | -0.34 (-0.82, 0.15), p=0.170 | p=0.028 |
|  | Study 2 | -0.44 (-1.12, 0.25), p=0.207 | 0.75 (0.06, 1.44), p=0.034 | p=0.004 | -0.34 (-1.03, 0.35), p=0.329 | 0.72 (0.03, 1.41), p=0.042 | p=0.012 |
|  | Studies 1&2 combined | -0.45 (-0.88, -0.03), p=0.038 | 0.24 (-0.19, 0.67), p=0.277 | p=0.006 | -0.44 (-0.87, -0.01), p=0.047 | 0.18 (-0.26, 0.61), p=0.43 | p=0.017 |
| Perceived size of computer images (at 24 hour follow up, adjusted for pre-task rating) (N=84) | Study 2 | 0.12 (-0.18, 0.42), p=0.413 | -0.47 (-0.77, -0.18), p=0.002 | p<0.001 | 0.17 (-0.13, 0.47), p=0.271 | -0.47 (-0.76, -0.18), p=0.002 | P<0.001 |
| Perceived own size (at 24 hour follow up, adjusted for pre-task rating) (N=84) | Study 2 | -0.05 (-0.39, 0.49), p=0.823 | -0.27 (-0.71, 0.17), p=0.229 | p=0.311 | -0.05 (-0.49, 0.39), p=0.825 | -0.33 (-0.77, 0.11), p=0.143 | p=0.286 |
| Satisfaction with own size (at 24 hour follow up, adjusted for pre-task rating) (N=84) | Study 2 | -0.18 (-0.89, 0.53), p=0.616 | 0.70 (-0.002, 1.40), p=0.051 | p=0.032 | 0.002 (-0.69, 0.69), p=0.994 | 0.67 (-0.01, 1.35), p=0.053 | p=0.080 |

*(Linear Regression Analyses with Randomisation group as the categorical exposure variable)
