# Supplementary material for: Effects of exposure to bodies of different sizes on perception of and satisfaction with own body size: two randomized studies
Source: R Soc Open Sci. 2018 May 9;5(5):171387. doi: 10.1098/rsos.171387 (PMC5990741; doi:10.1098/rsos.171387)
Supplement: Table 3 [file rsos171387supp2.docx]

Table 3: Changes in perception of size of others, own size and satisfaction with own size after removing outliers (Studies 1 & 2)

|  |  | Unadjusted comparison with those adapted to normal weight images | | | Comparison with those adapted to normal weight images, adjusted for age, BMI and PHQ-9 score | | |
| --- | --- | --- | --- | --- | --- | --- | --- |
|  |  | Adapted to underweight images | Adapted to overweight images | Test of Trend across all groups | Adapted to underweight images | Adapted to overweight images | Test of Trend across all groups |
| Perceived size of computer images (post-task rating adjusted for pre-task rating) | Study 1 | 0.59 (0.29, 0.88), p<0.001 | -0.23 (-0.54, 0.07), p=0.126 | p<0.001 | 0.66 (0.35, 0.96), p<0.001 | -0.20 (-0.51, 0.12), p=0.213 | p<0.001 |
|  | Study 2 | 0.33 (0.03, 0.63), p=0.034 | -0.39 (-0.70, -0.07), p=0.017 | p<0.001 | 0.38 (0.08, 0.69), p=0.015 | -0.34 (-0.66, -0.03), p=0.033 | p<0.001 |
|  | Studies 1&2 combined | 0.47 (0.26, 0.68), p<0.001 | -0.32 (-0.54, -0.10), p=0.004 | p<0.001 | 0.52 (0.31, 0.73), p<0.001 | -0.30 (-0.52, -0.08), p=0.007 | p<0.001 |
| Perceived own size (post-task rating adjusted for pre-task rating) | Study 1 | 0.42 (0.09, 0.71), p=0.011 | -0.04 (-0.36, 0.27), p=0.793 | p=0.008 | 0.38 (0.05, 0.70), p=0.023 | -0.03 (-0.36, 0.30), p=0.871 | p=0.022 |
|  | Study 2 | 0.50 (0.09, 0.90), p=0.017 | -0.19 (-0.62, 0.23), p=0.364 | p=0.005 | 0.48 (0.07, 0.89), p=0.021 | -0.26 (-0.70, 0.17), p=0.228 | p=0.003 |
|  | Studies 1&2 combined | 0.43 (0.18, 0.68), p=0.001 | -0.12 (-0.38, 0.15), p=0.383 | p=0.001 | 0.40 (0.14, 0.65), p=0.002 | -0.14 (-0.41, 0.12), p=0.29 | p<0.001 |
| Satisfaction with own size (post-task rating adjusted for pre-task rating) | Study 1 | -0.54 (-0.97, -0.11), p=0.014 | -0.31 (-0.75, 0.13), p=0.164 | p=0.047 | -0.61 (-1.06, -0.17), p=0.007 | -0.33 (-0.79, 0.12), p=0.150 | p=0.027 |
|  | Study 2 | -0.48 (-1.15, 0.20), p=0.161 | 0.57 (-0.13, 1.28), p=0.110 | p=0.014 | -0.39 (-1.08, 0.30), p=0.262 | 0.54 (-0.18, 1.25), p=0.140 | p=0.043 |
|  | Studies 1&2 combined | -0.42 (-0.83, 0.00), p=0.050 | 0.18 (-0.25, 0.61), p=0.41 | p=0.017 | -0.43 (-0.84, -0.07), p=0.046 | 0.10 (-0.33, 0.54), p=0.64 | p=0.034 |
| Perceived size of computer images (at 24 hour follow up, adjusted for pre-task rating) | Study 2 | 0.13 (-0.15, 0.42), p=0.353 | -0.56 (-0.85, -0.27), p<0.001 | P<0.001 | 0.17 (-0.11, 0.46), p=0.234 | -0.56 (-0.85, -0.27), p<0.001 | p<0.001 |
| Perceived own size (at 24 hour follow up, adjusted for pre-task rating) | Study 2 | 0.11 (-0.32, 0.55), p=0.605 | -0.33 (-0.77, 0.11), p=0.143 | p=0.127 | 0.01 (-0.43, 0.44), p=0.973 | -0.40 (-0.85, 0.04), p=0.075 | p=0.117 |
| Satisfaction with own size (at 24 hour follow up, adjusted for pre-task rating) | Study 2 | -0.23 (-0.96, 0.50), p=0.530 | 0.73 (-0.01, 1.47), p=0.054 | p=0.028 | -0.03 (-0.74, 0.69), p=0.940 | 0.74 (0.02, 1.47), p=0.044 | p=0.058 |

*(Linear Regression Analyses with Randomisation group as the categorical exposure variable)
